# Copper Dyshomeostasis, Redox Buffering and Immune Aging Converge on Cuproptosis in Age-Related Diseases

**DOI:** 10.3390/antiox15030353

**Published:** 2026-03-11

**Authors:** Yubin Jin, Keyu Lu, Yang Yang

**Affiliations:** Key Laboratory of Pathobiology (Yanbian University), State Ethnic Affairs Commission, Yanji 133000, China; jyb1985635005@outlook.com (Y.J.); lukeyu8687@163.com (K.L.)

**Keywords:** cuproptosis, copper homeostasis, redox buffering, oxidative stress, glutathione, mitochondrial metabolism, protein lipoylation, iron sulfur clusters, immunosenescence, inflammaging

## Abstract

Cuproptosis is a copper-dependent form of regulated cell death that is triggered when intracellular copper handling is perturbed and mitochondrial metabolism becomes the primary site of damage. Aging provides a biological context for this process because copper trafficking shifts, mitochondrial quality control and proteostasis decline, and immune function is remodeled toward immunosenescence with persistent low-grade inflammation. These age-associated changes can weaken antioxidant buffering, reshape labile copper pools, and lower the threshold at which copper stress is converted into mitochondrial proteotoxic injury. In parallel, inflammaging-related cytokines and NF-κB programs can alter copper import, export, and sequestration, while impaired efferocytosis prolongs danger signaling, creating feedforward loops that sustain tissue injury. In this review, we summarize the molecular features that distinguish cuproptosis from other death programs and discuss how redox buffering capacity, copper transport machinery, and mitochondrial metabolic state jointly determine cuproptosis sensitivity during aging. We then examine disease contexts in which these pathways are plausibly relevant, including hereditary copper-handling disorders and age-related neurodegenerative, cardiovascular, metabolic, and musculoskeletal disorders. Finally, we discuss key knowledge gaps and experimental priorities for interpreting cuproptosis-related signals in aged tissues, with emphasis on how copper handling, mitochondrial state, and immune remodeling jointly shape disease phenotypes.

## 1. Introduction

Physiological aging results in gradual physiological deterioration and disturbed homeostasis in virtually all organs. One of these is the immune system, which exhibits specific immune remodeling with loss of functional immunity (immunosenescence) and chronic low-grade inflammation (inflammaging) [1]. Immunosenescence is characterized by malfunction of immune organs (thymus, spleen), loss of production of naïve T cells and B cells, and defective function of innate cells (e.g., macrophages and neutrophils) [2]. Inflammaging results from continuous inflammatory insults (senescent cell secretions, metabolic disturbances, and gut dysbiosis) and contributes to tissue injury. Both conditions lead to an imbalance in the immune system that leads to infection susceptibility, inadequate vaccine responses, frailty, and other age-related disorders [3]. Cuproptosis has recently been recognized as a novel copper-induced regulated cell death. Unlike apoptosis, necroptosis or ferroptosis, cuproptosis is specifically induced by elevated levels of intracellular copper. Aging, immune dysfunction, and copper dyshomeostasis have pathological significance. An essential trace metal, copper facilitates numerous immunometabolic processes, including as a cofactor for phagocyte enzymes and T cell function. Clinically, copper deficiency or overload is detrimental to immunity; copper deficiency leads to impaired neutrophil killing and lymphocyte proliferation, while excessive intracellular copper induces cuproptotic mitochondrial damage [4]. Therefore, in aged subjects, the triad of immunosenescence (weaker host defense), inflammaging (chronic inflammation and oxidative stress), and metal imbalance may diminish the threshold of programmed cell death. Metabolically active cells (e.g., neurons, hepatocytes, and T cells) are particularly susceptible to copper-induced proteotoxic stress and mitochondrial damage. Together, these findings indicate that copper dyshomeostasis might worsen immunosenescence with aging and participate in disease pathology. With these links, there is good reason to explore cuproptosis in aging and immune remodeling. Elucidating aging-induced reprogramming of copper management and cell-death vulnerability could identify new avenues for intervention. As an example, copper manipulation using chelators/ionophores has been beneficial in cancer and neurodegeneration models, suggesting possibilities for repurposing in age-related conditions [5,6]. In this vein, this review discusses key cuproptosis mechanisms and regulation, immunometabolic regulation during aging, and therapeutic opportunities across age-related diseases. However, existing reviews have not fully addressed the unique challenges posed by aging biology, such as progressive copper imbalance, diminished redox buffering capacity, immune system remodeling, and organ-specific microenvironmental changes. These factors collectively alter the threshold for copper-dependent mitochondrial stress, providing a distinctive perspective on the role of copper toxicity in aging and age-related diseases.

To explore these concepts further, we conducted a comprehensive literature search using PubMed and Web of Science for English-language papers published between January 2021 and January 2026. Our search focused on keyword combinations related to cuproptosis and copper handling (e.g., copper homeostasis, FDX1, protein lipoylation, Fe-S biology, and mitochondria) in conjunction with aging and disease terms (e.g., immunosenescence, inflammaging, neurodegeneration, cardiometabolic disease, musculoskeletal aging, and cancer). In addition to database searches, we complemented our review with citation tracking from key primary studies. We prioritized works that defined copper-centric mechanisms and included measurable mitochondrial or cuproptosis-relevant endpoints, incorporating human evidence where available, alongside data from animal and cellular models. Earlier studies were cited selectively, primarily to establish foundational copper transport or mitochondrial concepts.

Therefore, this review places copper toxicity within the aging immune framework for the first time and organizes the evidence around three key transformation issues: how age-related immune states regulate copper handling and susceptibility, how organ microenvironments modulate copper toxicity sensitivity in major age-related diseases, and which treatment strategies remain feasible under elderly safety constraints.

## 2. Physiological Functions, Mechanism of Cuproptosis and Its Regulation in Aging

### 2.1. Physiological Functions and Homeostasis Regulation of Copper

Copper is an essential trace element that has an important role as a cofactor for many metabolic enzymes, as well as the ability to become toxic at higher intracellular concentrations [1]. Copper is required for mitochondrial oxidative phosphorylation (OXPHOS) and energy metabolism because it serves as an essential cofactor for cytochrome-c oxidase (CcO) in the electron transport chain. In order to balance the need for copper and avoid toxicity, cells have developed complex regulatory processes. High-affinity copper transporter 1 (CTR1; SLC31A1) is a high-affinity importer of dietary copper. Once inside the cell, copper is delivered by copper chaperone proteins like antioxidant protein 1 (ATOX1) and cytochrome c oxidase copper chaperone (COX17) to distinct sites within the cell (e.g., delivery to mitochondria for enzyme metallation) [7]. At the same time, cellular chelators, including glutathione (GSH) and metallothioneins, sequester any excess free copper, while copper-exporting ATPases (ATP7A/B) export excess copper out of the cytosol [8]. These mechanisms coordinate to control the level of free copper within the cytosol and protect cellular function.

Dysregulations of copper homeostasis cause severe alterations in cellular metabolism and cell fate, both in cases of copper excess and deficiency. In response to copper overload, intracellular surplus copper triggers the formation of reactive oxygen species (ROS) and adaptive changes in copper trafficking, including deregulated uptake via CTR1 and adaptive redistribution/upregulation of ATP7A/B-mediated export, resulting in enhanced copper uptake and intracellular copper load and enhanced cuproptosis-related signaling [9]. Copper deficiency, usually as a result of defective uptake via CTR1, leads to the dysfunction of the mitochondrial electron transport chain (particularly CcO activity). The cell experiences a progressive energy crisis due to decreased ATP generation and the failure of the tricarboxylic acid (TCA) cycle flux.

Importantly, both copper excess and deficiency lead to cellular dysfunction, making cells more vulnerable to cell death and tissue damage.

### 2.2. Discovery and Distinction of Cuproptosis

Cuproptosis is a recently characterized copper-mediated regulated cell death (RCD) that is initiated and propagated by elevated levels of intracellular copper, originally reported by Tsvetkov et al. [10]. Unlike classical cell death modes such as apoptosis, necroptosis, pyroptosis, or ferroptosis, cuproptosis is uniquely activated by copper ions and proceeds via its own mechanism [11]. Crucial support for cuproptosis has emerged from experiments involving copper ionophores (e.g., elesclomol, disulfiram). The cell death observed with these compounds can be selectively prevented by copper chelators but not by apoptosis, ferroptosis, or other death pathway inhibitors, indicating a new mechanism [12]. For example, while apoptosis requires caspase-mediated DNA fragmentation, cuproptosis does not, and whereas ferroptosis depends on lipid peroxidation, cuproptosis does not. Other death pathway inhibitors have not reversed the phenotype under the experimental conditions used, yet copper chelation reduces toxicity (e.g., ammonium tetrathiomolybdate (TTM)), highlighting copper dependency [13]. Taken together, these data establish cuproptosis as a new RCD pathway featuring a copper-driven initiation and execution process.

### 2.3. Core Molecular Mechanisms and Regulation

**Figure 1:**
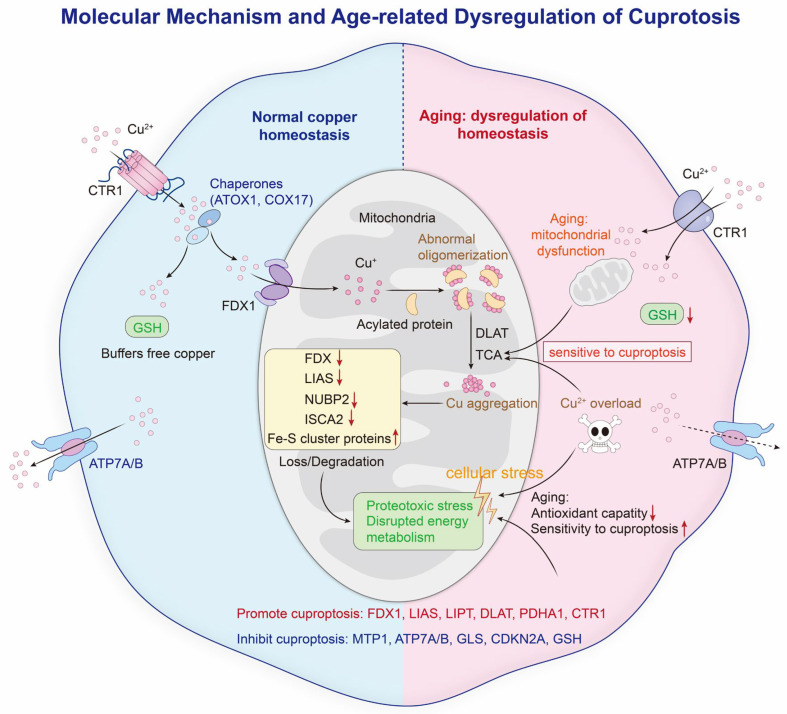
Molecular mechanism and age-related dysregulation of cuproptosis. Copper enters cells via CTR1 and is distributed by chaperones, buffered by GSH, and exported by ATP7A/ATP7B to maintain homeostasis. Cuproptosis is initiated when mitochondrial Cu^+^ binds lipoylated TCA cycle proteins such as DLAT, causing protein aggregation, Fe-S protein loss, and proteotoxic stress. Aging disrupts copper handling and mitochondrial function and lowers antioxidant capacity, thereby increasing sensitivity to cuproptosis. Abbreviations: CTR1, copper transporter 1; GSH, glutathione; ATP7A, ATPase copper transporting alpha; ATP7B, ATPase copper transporting beta; Fe-S, iron–sulfur; Cu^+^, cuprous ion; Cu^2+^, cupric ion.

Activation of cuproptosis requires perturbation of copper homeostasis and mitochondrial metabolism. Increased copper entry (via CTR1) but decreased exit (via ATP7A/B) leads to copper buildup inside cells, especially in mitochondria, setting the stage for cuproptosis. On the other hand, induction of ATP7A/B increases copper export, reducing intracellular copper levels and blocking cuproptosis [14]. Inside mitochondria, ferredoxin 1 (FDX1) reduces Cu^2+^ to Cu^+^, which can then bind to the lipoylated sites of several important TCA-cycle enzymes, mainly the E2 component (dihydrolipoamide S-acetyltransferase, DLAT) of the pyruvate dehydrogenase complex [15]. Importantly, this toxic interaction requires the lipoic acid modification of these enzymes, which is made by the lipoylation pathway (enzymes such as lipoic acid synthetase (LIAS) and lipoyltransferase 1 (LIPT1)) [16]. Lipoylated proteins bound by copper undergo abnormal oligomerization and aggregation to form insoluble protein aggregates, causing mitochondrial proteotoxic stress and impairing energy metabolism [11]. In parallel, excess copper also interferes with biogenesis and maintenance of mitochondrial iron–sulfur (Fe-S) cluster proteins (e.g., NUBP2, CIAPIN1, and ISCA2) [17]. Loss of these crucial cofactors incapacitates respiratory complexes and other Fe-S-containing metabolic enzymes, leading to defective electron transfer and ATP synthesis. Together, mitochondrial proteotoxic stress and Fe-S cluster depletion lead to dysfunction and the fatal collapse of mitochondrial structure and metabolism, leading to irreversible cuproptosis (Figure 1).

Notably, cuproptosis specifically takes place in cells with functional mitochondrial TCA-cycle activity and functional protein lipoylation machinery and is not a form of cell death mediated by ROS (e.g., ferroptosis) or GSH depletion-induced cell death [18]. Several factors influence cellular susceptibility to cuproptosis. Inducers of cuproptosis are FDX1, lipoylation enzymes (LIAS and LIPT1), and lipoylated TCA-cycle proteins (such as DLAT and PDHA1). On the other hand, inhibitors are copper exporters ATP7A/B and metal-responsive transcription factor metal–regulatory transcription factor 1 (MTF1) (which assists in copper sequestration and limiting copper availability) [17] (Table 1).

### 2.4. Regulation and Alterations of Cuproptosis During Aging

Aging can impair copper homeostasis across uptake, distribution, trafficking, and excretion, which may shift cells toward copper accumulation and increased susceptibility to cuproptosis. In fact, many aged tissues show abnormal copper redistribution and accumulation, such as increased free copper and ceruloplasmin in serum, and such dyshomeostasis correlates with inflammaging, a feature of aging [21]. At the same time, age-related mitochondrial dysfunction and antioxidant defense decline increase cellular vulnerability to copper-induced proteotoxic stress and cuproptosis [21]. Therefore, aged cells are more susceptible to copper-driven mitochondrial dysfunction and loss of metabolic resilience than young cells.

Similarly, excess copper accumulation in aged cells has been associated with increased ROS generation and aberrant protein aggregation. These changes may activate stress-response pathways (e.g., nuclear factor erythroid 2-related factor 2 (Nrf2) and p38 mitogen-activated protein kinase (MAPK)) [22], increasing cuproptosis risk and severity. Several aging pathways are likely to influence susceptibility, such as chronic oxidative stress, proteostasis dysregulation, mechanistic target of rapamycin (mTOR) signaling dysregulation, reductions in mitochondrial biogenesis, and altered regulation of metal-responsive factors like MTF1 [23]. Current data suggest that aging tissues may have a compromised ability to export copper, possibly through changes in ATP7A/B expression or function alongside reduced antioxidant defenses. This may lower the threshold for copper-triggered mitochondrial stress and increase cuproptosis susceptibility. The extent and universality of ATP7A/B downregulation during human aging requires validation using prospectively acquired age-stratified samples [23,24].

Intracellular antioxidants like GSH provide limited protection through chelation of labile copper and redox homeostasis and by potentially inhibiting lipoylated protein aggregation to delay cuproptosis onset [25,26]. Copper dyshomeostasis may also affect redox homeostasis. For instance, elevated copper levels may induce autophagic clearance of antioxidant enzymes, including glutathione peroxidase 4 (GPX4), an important enzyme that protects cells from lipid peroxidation [27,28]. This observation suggests a potential interplay between cuproptosis and ferroptosis, whereby simultaneous copper toxicity and lipid peroxidation could synergistically exacerbate tissue damage in aging.

## 3. Age-Related Immune Changes

Aging alters immune cell turnover and skews inflammatory signaling, so many tissues operate under a higher baseline of immune activation. In this setting, copper stress is less effectively buffered: uptake pressure can rise, export capacity may be constrained, and oxidative conditions reduce GSH-dependent protection. As a result, microenvironments in older tissues may be more prone to conditions under which mitochondrial copper stress becomes damaging. Once injury occurs, clearance is often slower. Declining efferocytosis and weakened pro-resolving programs allow debris and danger signs to persist, sustaining innate activation and reinforcing a local loop between inflammation and copper stress.

### 3.1. Immunosenescence

#### 3.1.1. The Concept and Main Characteristics of Immunosenescence

Immunosenescence refers to the age-related decline and remodeling of both innate and adaptive immunity, and it is a key factor underlying older individuals’ vulnerability to diseases [29,30]. This yields a mixed phenotype in which impaired antimicrobial defense coexists with chronic low-grade inflammation, jointly shaping disease susceptibility across organs [31]. Adaptive immunity is remodeled with age as thymic involution curtails naïve T cell production and narrows T cell receptor (TCR) diversity, shifting the repertoire toward memory and terminally differentiated subsets with limited proliferative capacity and reduced helper function [32]. In parallel, B cell output and germinal center performance decline, restricting antibody diversification and weakening vaccine durability [33]. Age-related dysfunction in macrophage metabolism and GSH synthesis can also constrain copper buffering and favor intracellular copper accumulation, predisposing inflamed tissues to copper-triggered stress [32]. Across lineages, recurrent senescence/exhaustion features contribute to an immune response that can be simultaneously pro-inflammatory and immunosuppressive, progressively reshaping tissue microenvironments [34].

#### 3.1.2. Molecular and Cellular Underpinnings

Immunosenescence results from defects that converge at the levels of hematopoiesis, genome maintenance, antigen-driven remodeling, and cellular metabolism. Hematopoietically, aging hematopoietic stem cells gradually fail to maintain long-term quiescence and display a myeloid-biased differentiation profile that compromises the reconstitution of naive T- and B-lymphocytes and limits long-term renewal potential [35]. Meanwhile, replicating immune cells suffer replicative stress, and telomere shortening and DNA damage trigger canonical responses, such as adipose tissue macrophage (ATM)-p53-p21 pathways and nuclear factor kappa B (NF-κB)-linked transcriptional programs that stabilize cell-cycle exit and loss of function [36].

These programs are consolidated by epigenetic remodeling and by persistent stress inputs that promote redox and mitochondrial dysfunction, which can favor a chronic inflammatory baseline [32]. Importantly, NF-κB-mediated inflammatory signaling has been associated with dysregulated expression of copper-handling factors, potentially linking immune aging with copper-induced proteotoxic stress implicated in cuproptosis [37]. Although substantial interindividual and lineage-specific heterogeneity exists, recurrent phenotypes include exhaustion/senescence markers and restricted functional reserve, which complicate assessment but define tractable intervention points [34].

### 3.2. Inflammaging

#### 3.2.1. Concept and Drivers

Inflammaging describes a chronic, low-grade, sterile, systemic inflammatory state that becomes increasingly prevalent with advancing age and is commonly reflected by modest but sustained elevations of circulating inflammatory mediators, including interleukin-6 (IL-6), tumor necrosis factor-α (TNF-α), and C-reactive protein (CRP) [38]. In contrast to acute inflammation, which is typically high amplitude and self-limited, inflammaging is lower intensity yet persistent, and it can accumulate over time as immune and tissue homeostatic mechanisms progressively decline. Multiple, partially overlapping inputs can sustain this state. These include persistent antigenic stimulation, increased translocation of microbial products associated with impaired barrier integrity, cumulative cell and tissue damage that generates endogenous danger signals, and age-related impairment of efferocytosis and debris clearance. Together, these factors promote sustained engagement of innate immune sensing pathways and a failure to fully resolve inflammatory signaling, thereby maintaining a baseline inflammatory tone in older individuals [39]. Clinically, inflammaging is tightly associated with multimorbidity and functional decline and is increasingly recognized as a permissive background that can lower stress resilience in late life.

#### 3.2.2. Molecular Mechanisms of Inflammaging and the Associated Tissue and Cellular Damage

Inflammaging is maintained by partially overlapping processes that shift immune signaling toward a higher basal inflammatory tone. Thymic involution and reduced naive T cell input occur in parallel with heightened activity of inflammatory signaling modules, including NF-κB and mTOR, and increased propensity for inflammasome activation, particularly NLR family pyrin domain containing 3 (NLRP3), which collectively weakens immune surveillance while raising the inflammatory set point [39]. Persistent antigenic stimulation and continuous generation of endogenous danger signals can keep pattern recognition receptor pathways engaged and prolong cytokine production over time [40,41]. A second major driver is the age-associated accumulation of senescent cells, whose senescence-associated secretory phenotype (SASP) reinforces local and systemic inflammation and can propagate dysfunction through paracrine loops [31].

Crosstalk between inflammatory signaling and copper homeostasis may further shape tissue susceptibility. Damage-associated molecular pattern (DAMP)-driven Toll-like receptor 4 (TLR4)/NF-κB signaling has been linked to changes in copper-handling pathways, including altered CTR1-associated copper uptake capacity in inflammatory contexts. Under copper dyshomeostasis, expansion of labile copper can make copper-dependent mitochondrial stress more likely and may further intensify DAMP-driven inflammatory signaling [40]. Whether this sequence represents a major driver of tissue damage in aging organisms remains to be validated in age-stratified in vivo models. Over time, sustained inflammation can impair reparative pathways such as autophagy, increase oxidative and mitochondrial stress, and facilitate maladaptive wound healing responses, culminating in fibrosis and organ dysfunction [42]. SASP components can also amplify damage beyond the originating site by inducing dysfunction in adjacent cells and expanding the footprint of senescence [43].

### 3.3. System-Level Consequences and Disease Susceptibility

Immunosenescence and inflammaging impact host defense and tissue homeostasis, increasing vulnerability to age-related diseases. Sustained low-grade inflammatory signaling has been linked to cardiovascular disease, metabolic disorders including type 2 diabetes, neurodegenerative diseases including Alzheimer’s disease, osteoporosis, sarcopenia, and cancer [40,44]. While the inflammatory response is limited, its duration on a timescale of years may lead to cumulative biological attrition and gradual loss of function across tissues [31].

In parallel, declines in both innate and adaptive immune function reduce protection against infection and weaken vaccine effectiveness and durability in older populations [45,46]. Immune aging also compromises tumor immunosurveillance through reduced cytotoxic activity and increased inhibitory signaling, creating a tumor-permissive environment and contributing to weaker responses to immunotherapy in older patients [47]. Across organ systems, chronic inflammatory signaling provides a permissive background for degenerative pathology, including atherosclerotic progression and neuroinflammatory trajectories [48]. Inflammaging is also associated with musculoskeletal decline and has been linked to chronic inflammatory and autoimmune conditions, including rheumatoid arthritis, osteoporosis, and sarcopenia [1].

**Figure 2:**
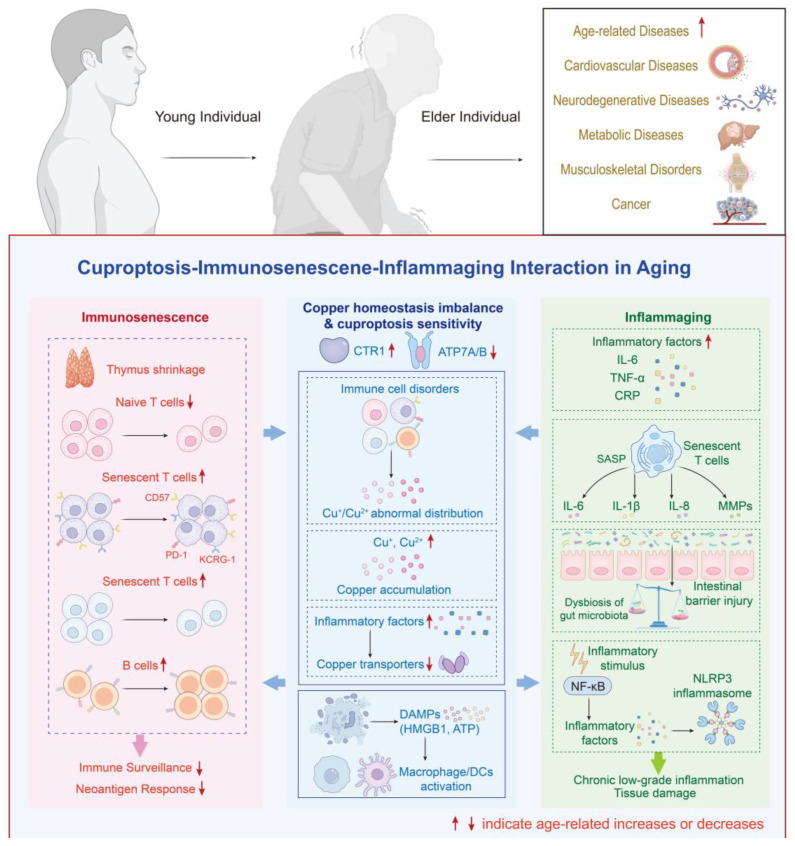
Cuproptosis, immunosenescence, and inflammaging interaction in aging. Immunosenescence and inflammaging reshape copper transport, inflammatory signaling, and cellular stress responses in aging tissues. These changes promote abnormal copper distribution and accumulation, increase cuproptosis sensitivity, and amplify DAMP-driven activation of innate immune cells. The schematic also summarizes major disease categories that increase with aging. Abbreviations: DAMP, damage-associated molecular pattern; immunosenescence, age-associated decline in immune function; inflammaging, chronic low-grade inflammation during aging.

Importantly, inflammatory consequences of the aged tissue microenvironment may intersect with intracellular copper handling, thereby influencing susceptibility to copper-dependent proteotoxic stress. Pro-inflammatory cytokines and NF-κB-linked programs are tightly coupled to cellular stress adaptation and metabolic rewiring in aging tissues, and this inflammatory milieu can coexist with shifts in copper handling that favor copper-dependent mitochondrial stress. In this context, copper accumulation may lower the threshold for mitochondrial proteotoxic injury that aligns with cuproptosis biology (Figure 2).

Although a direct, subset-resolved causal chain linking immunosenescence or inflammaging to copper transporter remodeling and altered cuproptosis sensitivity is still largely unavailable, a small number of recent functional studies provide direct anchors for key steps of this connection in DAMP-dominant innate immune contexts. In inflammatory macrophages, a mitochondria-associated reactive Cu(II) pool has been shown to sustain inflammatory metabolic and epigenetic programming, and disrupting this copper signaling axis suppresses inflammation in vivo, demonstrating that danger-associated inflammatory states can actively re-route intracellular copper rather than merely correlate with it [49]. Copper can also act as an intracellular signal that tunes innate immune sensing through ALPK1 kinase, placing copper regulation within pathways aligned with PRR and DAMP-linked danger responses [50]. Consistent with these direct links, in LPS-stimulated macrophages and an in vivo inflammatory model, copper chelation attenuates inflammation while shifting expression of key copper transport genes, including ATP7A and CTR1, together with cuproptosis-associated molecular readouts, supporting the idea that inflammatory activation can be coupled to transporter remodeling and a shifted threshold for copper-dependent mitochondrial stress that is compatible with cuproptosis sensitivity [51]. Taken together, these findings suggest a plausible intersection between immunosenescence, inflammaging, and copper transporter remodeling that could influence cuproptosis sensitivity in certain immune subsets. But the specific regulatory mechanism still needs further in-depth analysis in the future.

## 4. Organ-Specific Pathological Manifestations of Cuproptosis in Aging

### 4.1. Neurodegenerative Diseases

Neurodegenerative diseases emerge from a convergence of neuronal metabolic vulnerability and age-dependent failure of immune-mediated tissue maintenance. In the aging central nervous system, barrier dysfunction and altered glial homeostasis can change regional copper distribution, while chronic microglial activation sustains a cytokine-rich environment that perturbs redox buffering and mitochondrial quality control. Within neurons, cuproptosis may be engaged when copper accumulates in mitochondria that retain an active TCA cycle and a lipoylation-competent protein network. Under these conditions, mitochondrial copper stress may contribute to neuronal metabolic failure and loss of viability [52]. Age-related immune changes can amplify this primary insult. Inefficient clearance of damaged neurites and dying cells allows damage-associated molecular patterns to persist, reinforcing microglial priming and sustaining a feed-forward loop between cell death and neuroinflammation [53].

Multiple neurodegenerative phenotypes intersect with copper-dependent proteostasis and mitochondrial stress. In Alzheimer’s-related pathology, copper can interact with amyloid beta and tau species and may influence aggregation and synaptic toxicity, while parallel mitochondrial impairment can increase cuproptosis susceptibility [54,55]. In Parkinson’s-related pathology, copper imbalance co-occurs with alpha synuclein misfolding and mitochondrial dysfunction, consistent with a model in which copper-driven stress promotes both proteopathic spread and neuronal loss [56]. In amyotrophic lateral sclerosis, altered copper handling, including pathways linked to SOD1 biology, can contribute to motor neuron injury, and copper-targeted interventions have been investigated as neuroprotective approaches in experimental settings [57]. Beyond chronic neurodegeneration, acute ischemic injury can also create local copper dysregulation and mitochondrial damage that may engage cuproptosis-related mechanisms, linking copper burden to secondary inflammatory injury after stroke [52,58].

### 4.2. Cardiovascular Diseases

Cardiovascular aging is characterized by progressive mitochondrial stress, altered redox signaling, endothelial dysfunction, and a chronic inflammatory tone that may reshape copper handling and cuproptosis sensitivity [59]. Copper is indispensable for multiple cardiac enzymes, yet aging can shift copper toward a more reactive pool through changes in transport, binding, and compartmentalization. When labile copper increases within cardiomyocyte or vascular-cell mitochondria, copper stress can impair respiratory capacity and promote cell injury, thereby contributing to age-associated remodeling. Age-associated myeloid bias and inflammasome activation can amplify tissue damage and impair repair, while dying cells provide signals that further recruit inflammatory leukocytes, sustaining maladaptive remodeling [60].

In atherosclerosis, plaque progression depends on endothelial injury, lipid accumulation, and inflammatory immune cell activity. Copper accumulation within lesions can intensify oxidative modification of lipids and proteins and may increase the susceptibility of plaque resident endothelial cells, macrophages, and smooth muscle cells to copper-dependent mitochondrial injury. Cell death within plaques can release proinflammatory mediators and debris that are incompletely cleared in aging, thereby amplifying inflammatory circuits and destabilizing lesions [60]. The idea that copper modulation could impact lesion biology has been demonstrated experimentally and warrants exploration of copper chelation as an adjunctive approach in atherosclerotic disease [61].

Copper, mitochondria, and immunity also come together in the setting of myocardial ischemia and reperfusion injury. Ischemia reprograms cardiac metabolism, reperfusion produces abrupt oxidative stress, and local copper redistribution can accompany this transition. Mitochondrial injury can, therefore, engage copper-sensitive proteotoxic pathways and promote cardiomyocyte death, while the ensuing sterile inflammation influences infarct expansion and remodeling [62]. Changes in cardiac copper transporters during injury suggest that copper trafficking is dynamically regulated under stress, and copper-targeted interventions have been evaluated for their ability to attenuate inflammation and preserve function in experimental and clinical settings [63].

In heart failure and metabolic cardiomyopathies, chronic metabolic stress can maintain a background of mitochondrial dysfunction and inflammation that increases vulnerability to copper-dependent injury. In these settings, altered expression of cuproptosis-related genes and copper transport machinery has been associated with disease progression, supporting the need for mechanistically informed biomarkers to identify patients most likely to benefit from copper modulation [64,65].

### 4.3. Metabolic Diseases

Metabolic diseases of aging, including obesity, type 2 diabetes, and fatty liver disease, are shaped by tissue-specific copper imbalances, mitochondrial dysfunction, and chronic inflammation. Such conditions provide a metabolic environment whereby cuproptosis susceptibility may rise in hepatocytes, adipocytes, beta cells, and cardiometabolic tissues via cumulative copper stress and mitochondrial susceptibility [66]. Insulin resistance and high-fat feeding may impact intestinal uptake, liver sequestration, and redistribution, causing copper insufficiency in certain compartments and copper excess in other compartments [67,68].

Mitochondrial dysfunction caused by inflammatory macrophage infiltration and oxidative stress in obesity and fatty liver disease may create a situation where any remaining TCA capacity is more heavily relied upon, which may render cells more vulnerable to copper-induced proteotoxic stress in mitochondria. Cuproptosis-related genes’ transcriptomic signatures have been linked to fatty liver phenotypes, suggesting that mitochondrial stress mediated by copper may play a role in disease progression in predisposed patients [69].

In type 2 diabetes, chronic hyperglycemia, ROS, and advanced glycation products can damage mitochondria and alter protein modification pathways, potentially sensitizing pancreatic beta cells to copper-dependent injury. Concurrent immune dysregulation, including persistent low-grade cytokine signaling, can further impair beta cell survival and systemic metabolic control [70,71].

In metabolic-associated steatotic liver disease, copper dyshomeostasis is often better understood as compartmental remodeling rather than a uniform increase or decrease. Shifts in copper trafficking and buffering may lower the threshold for mitochondria-centered proteotoxic stress, especially when hepatocytes face sustained lipotoxic and inflammatory pressure and have limited respiratory reserve [72]. Under these conditions, copper-linked stress could be more likely to destabilize mitochondrial metabolism, consistent with observations that cuproptosis-related genes track with steatosis-associated features and markers of more advanced disease.

Cuproptosis-associated states may also extend beyond classic liver–adipose axes into metabolic–endocrine and cardiorenal complications. In polycystic ovary syndrome, reported cuproptosis-related signatures coincide with the enrichment of lipid-handling and atherosclerosis-relevant pathways, suggesting a plausible connection between copper-linked mitochondrial vulnerability and cardiometabolic risk features [73]. In diabetic kidney disease, cross-program comparisons do not consistently position cuproptosis as the dominant global cell death route, yet single-cell analyses indicate that cuproptosis activity can be concentrated in specific epithelial subtypes, highlighting cell-type-restricted vulnerability that may be obscured in bulk tissue assessments [74]. Together, these observations support a context-dependent model in which copper distribution, mitochondrial metabolic reliance, and inflammatory tone jointly determine whether cuproptosis functions as a peripheral signal or a pathogenic amplifier across metabolic disease tissues.

### 4.4. Age-Related Musculoskeletal Disorders

Musculoskeletal diseases of aging, including osteoarthritis and osteoporosis, are the consequence of accumulated mechanical strain, cellular senescence, mitochondrial dysfunction, and an inflammatory-biased joint/bone immune environment. In this setting, aberrant copper metabolism may promote mitochondrial stress and cell death in chondrocytes and osteoblast lineage cells through cuproptosis-linked processes and may contribute to tissue breakdown [75,76].

Synovial inflammation and oxidative stress in osteoarthritis may modify copper transporter expression in cartilage cells, leading to elevated intracellular labile copper and sensitization of mitochondria to copper-induced proteotoxic stress. Synovial macrophages and other immune cells influence cytokine exposure and debris removal, and aging decreases the effectiveness of resolution, leading to extended inflammatory tissue damage and death signaling [77,78].

The aging process causes a disruption in bone homeostasis due to an increase in inflammatory cytokine signaling, coupled with a reduction in anabolic signals that would otherwise synchronize osteoblast and osteoclast function. Both copper dyshomeostasis and cytokines of immune origin can compromise osteoblast mitochondrial health and matrix synthesis, as well as stimulate osteoclastogenesis [79].

### 4.5. Inherited Disorders of Copper Handling

Inherited disorders of copper handling are useful to include here because the direction of copper misdistribution is genetically fixed, which makes copper-linked mitochondrial vulnerability easier to interpret than in multifactorial age-related diseases. Menkes disease is an X-linked condition caused by ATP7A loss-of-function [80]. Defective copper export and systemic distribution lead to profound copper deficiency with markedly reduced copper delivery to the brain. A consistent downstream consequence is impaired mitochondrial respiration, including reduced CcO function and limited respiratory capacity, illustrating how copper scarcity can cap bioenergetic output in highly dependent tissues [81] (Table 2). CTR1 deficiency provides a complementary example from the import side. Loss of CTR1, the major copper importer, produces severe intracellular copper depletion, rapid neurodevelopmental deterioration, and cellular evidence of mitochondrial dysfunction, reinforcing that adequate copper entry is a prerequisite for maintaining mitochondrial metabolism [6].

Copper-overload disorders sit at the opposite extreme and connect more directly to cuproptosis-relevant toxicity. In Wilson’s disease, ATP7B dysfunction impairs biliary copper excretion and drives progressive hepatic copper accumulation [82]. Sustained excess can focus stress on mitochondria and, in overload states, is consistent with the cuproptosis framework, in which copper interacts with lipoylated mitochondrial enzymes and perturbs Fe-S biology. MEDNIK syndrome (AP1S1) disrupts ATP7A/B trafficking and can be associated with hepatic copper accumulation, although direct evidence that cuproptosis is a primary driver remains limited [81].

### 4.6. Geriatric Oncology

Geriatric oncology is characterized not only through tumor-intrinsic changes but also by an aged host environment with immunosenescence, inflammaging, and disturbed metal metabolism. Many tumors show increased dependence on copper-related pathways that support proliferation, angiogenesis, and redox buffering, while aging can impair systemic copper regulation and shift copper toward a more bioactive pool [83]. These host and tumor features are consistent with heightened susceptibility to copper stress and may limit the efficiency with which tumor cell death is converted into durable antitumor immunity, but direct validation in geriatric cancer models remains limited [84].

At the tumor cell level, altered expression of copper import and export machinery can increase intracellular copper availability. When combined with high mitochondrial metabolic activity, this configuration may lower the threshold for cuproptosis. However, the immune context of the aged tumor microenvironment is often suppressive. Presentation of antigens may be less efficient, effector T cell activation may be diminished, and exhausted or regulatory phenotypes may prevail. As a consequence, tumor cell death may not reliably translate into sustained immune control in older hosts, even when immunogenic programs are engaged in principle [28,85].

In organ-specific discussions of cuproptosis, because aging tissues share broad stress programs, the most direct organ-adapted route is currently in the liver. The hepatic copper can be quantified in biopsy-based cohorts and linked to disease severity or progression in steatotic liver disease [86]. In cardiovascular disease, circulating copper phenotypes (e.g., serum copper) are supported by systematic evidence relating higher levels to myocardial infarction, stroke, and cardiovascular mortality, providing a practical validation layer beyond expression-only analyses [87]. In the kidney, DKD can move beyond transcriptomic inference by incorporating clinically tractable urinary biomarkers, including urinary ceruloplasmin within multi-marker panels, which can be integrated with renal tissue analyses and age stratification [88]. By contrast, in the brain, Alzheimer’s-related associations remain largely transcriptome-driven and are better strengthened by copper phenotyping, which enables patient stratification (including non-ceruloplasmin copper), and by proteomic/multi-omics anchoring [89]. Endocrine–metabolic contexts can similarly reduce reliance on expression-only inference by combining copper phenotyping with metabolic readouts across diabetes-related trajectories [90]. In reproductive disease, meta-analytic evidence supports altered circulating copper in PCOS, offering an accessible but still indirect layer that requires tissue-level validation in age-relevant models [91]. In musculoskeletal disorders such as osteoarthritis, trace-element meta-analyses combined with Mendelian randomization provide stronger support than transcriptomics alone, although execution-level confirmation in aged tissues remains necessary [92,93]. Accordingly, each organ section is stratified into direct versus indirect evidence to avoid overinterpreting shared aging stress programs as cuproptosis.

## 5. Therapeutic Strategies Targeting Cuproptosis in Age-Related Diseases

Therapeutic strategies that modulate copper to influence cuproptosis are currently most advanced in oncology, where copper demand and mitochondrial redox vulnerability can be therapeutically exploited. Tumors often exhibit increased copper flux and altered copper handling, which creates opportunities for interventions that either promote intracellular copper stress or restrict copper availability. Copper ionophores, including elesclomol and disulfiram, have shown anticancer activity across experimental models and selected disease contexts [94]. Importantly, cuproptosis is increasingly viewed not as a stand-alone cytotoxic mechanism but as a sensitizing strategy that can enhance the efficacy of combination therapies [95]. This framing becomes particularly relevant in older adults because age-associated decline in organ reserve and the high prevalence of multimorbidity tend to narrow the therapeutic window of systemic copper perturbation.

A major line of investigation is to integrate cuproptosis modulation into immunotherapy. In older patients, immunosenescence and chronic inflammatory remodeling can reduce baseline responsiveness to immune checkpoint inhibitors while simultaneously increasing susceptibility to treatment-related adverse events. Against this background, sensitizing approaches are attractive when they can increase tumor immunogenicity without imposing prolonged systemic stress. Preclinical studies suggest that cuproptosis-related mitochondrial stress can promote immunogenic signaling, strengthen antigen presentation, and improve immune cell penetration into the tumor microenvironment [96]. These observations are largely derived from oncology-focused systems and acute perturbation settings, and their generalizability to aging tissues with altered repair capacity and immune reserve remains uncertain. When combined with PD-1 or PD-L1 blockade, such priming has been associated with improved tumor control and greater durability of response in experimental settings [97]. For translation, the key issue is to define dosing and sequencing schemes that enhance immune activation while maintaining an acceptable safety profile, which is particularly important in geriatric settings. Practical regimen design in older adults should also account for baseline frailty, nutritional status, renal and hepatic reserve, and the frequent presence of polypharmacy that may influence copper handling and mitochondrial stress tolerance.

Alongside these oncology-driven approaches, it is important to highlight that the field already has clinically deployable tools that shift systemic copper exposure, even though they were developed for different indications. Several FDA-approved products are used for copper replacement, copper-input reduction, de-coppering strategies in defined contexts, or trace-element supplementation in parenteral nutrition (Table 3). These agents matter in an elderly-focused discussion because they are monitorable and titratable in routine care, and they, therefore, provide a practical framework for risk mitigation. In older adults, the consequences of overcorrection can be clinically meaningful. Excessive copper lowering may contribute to anemia, leukopenia, neuropathy, impaired wound healing, or reduced host defense, whereas excessive copper supplementation may aggravate oxidative stress in susceptible tissues. As a result, these products define realistic ranges for modulating bioavailable copper and support a risk-mitigation approach that prioritizes avoiding iatrogenic deficiency, monitoring organ function, and accounting for polypharmacy.

Beyond immune checkpoint combinations, cuproptosis modulation has also been discussed in the context of metabolic co-targeting. Many tumors display rewired energy metabolism, and tumors arising in aged tissues may show altered mitochondrial function and reduced metabolic flexibility. In this setting, combining copper ionophores with agents that constrain glycolysis or oxidative phosphorylation has been proposed to intensify metabolic stress and broaden vulnerability without requiring overly granular molecular subclassification [13,101]. Such metabolic co-targeting may also reshape an immunosuppressive milieu, including lactate-associated immune suppression, thereby complementing immunotherapy in selected contexts [102]. From a geriatric perspective, however, the feasibility of such combinations hinges on tolerability and cumulative toxicity. Older adults may have a narrower buffer against weight loss, fatigue, neuropathy, cytopenias, and organ dysfunction, and these considerations often dictate whether a mechanistically appealing combination can be delivered at effective intensity.

Copper depletion represents a distinct strategy that removes a growth-supportive resource in copper-addicted tumors rather than forcing copper-driven mitochondrial stress. Copper chelation has been linked to anti-angiogenic and anti-metastatic effects, and tetrathiomolybdate has progressed toward clinical evaluation in this direction [103,104]. Mechanistically, copper lowering may also influence stromal and immune components of the tumor microenvironment, potentially alleviating immune exclusion and improving sensitivity to immune-based therapy [16,105]. In elderly patients, the clinical challenge is to avoid drifting into functional copper deficiency. This is particularly relevant when baseline nutritional intake is marginal or when gastrointestinal absorption is impaired, and it argues for conservative dose titration with longitudinal surveillance of hematologic parameters, neurologic symptoms, and organ function.

**Figure 3:**
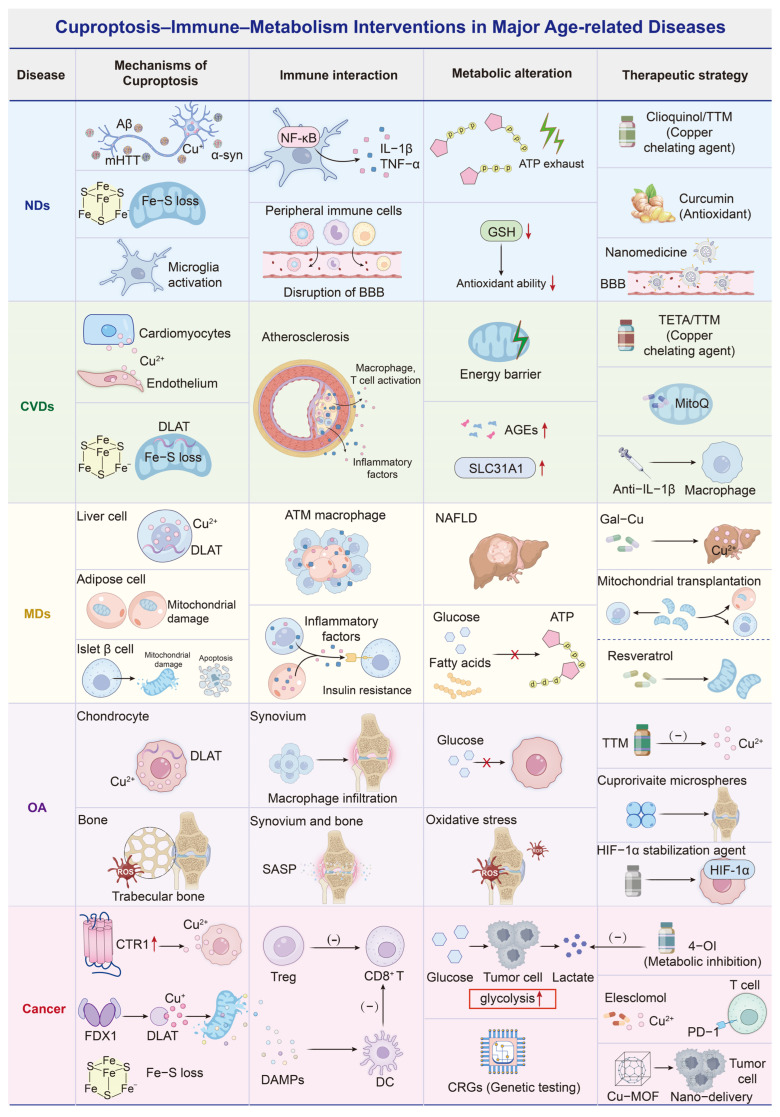
Cuproptosis, immune interaction, metabolic alteration, and interventions in major aging-related diseases. The figure integrates cuproptosis-related mechanisms with immune and metabolic features across neurodegenerative, cardiovascular, metabolic, and musculoskeletal diseases and cancer. Representative interventions are shown, including copper chelation and ionophore strategies, anti-inflammatory approaches, mitochondrial-targeted agents, and nanomedicine-based delivery concepts. Abbreviations: 4-OI, 4-octyl itaconate; Aβ, amyloid-β; AGEs, advanced glycation end products; ALS, amyotrophic lateral sclerosis; ATM, adipose tissue macrophage; BBB, blood–brain barrier; CRGs, cuproptosis-related genes; CTR1, copper transporter 1 (SLC31A1); Cu-MOF, copper-based metal-organic framework; CVDs, cardiovascular diseases; DAMPs, damage-associated molecular patterns; DC, dendritic cell; DLAT, dihydrolipoyl acetyltransferase; Fe-S, iron–sulfur; FDX1, ferredoxin 1; Gal-Cu, galactose-modified copper (delivery system/complex); GSH, glutathione; HIF-1α, hypoxia-inducible factor-1α; IL-1β, interleukin-1β; MDs, metabolic diseases; mHTT, mutant huntingtin; MitoQ, mitochondria-targeted ubiquinone; NAFLD, non-alcoholic fatty liver disease; NDs, neurodegenerative diseases; NF-κB, nuclear factor kappa B; OA, osteoarthritis; PD-1, programmed cell death protein 1; ROS, reactive oxygen species; SASP, senescence-associated secretory phenotype; TETA, triethylenetetramine; TNF-α, tumor necrosis factor-α; Treg, regulatory T cell; TTM, tetrathiomolybdate.

Outside oncology, the therapeutic framing should shift. For most non-cancer age-related diseases, the goal is functional preservation rather than cell elimination. In older adults, the near-term goal is more often to limit maladaptive copper stress and preserve mitochondrial function than to deliberately induce cuproptosis. Across neurodegenerative disorders, copper-binding or redistribution strategies have been associated with neuronal protection and improved functional outcomes in experimental settings, though results depend on disease stage and tissue context [57]. In cardiovascular models, copper chelation has been linked to reduced vascular inflammation and improved outcomes in atherosclerosis, ischemia–reperfusion injury, and cardiomyopathy [59,61,63]. In metabolic diseases, both supplementation and chelation have been described as beneficial depending on organ-specific copper needs and disease severity, again implying that baseline copper status and comorbid burden are likely to shape response [67,69]. Copper accumulation has also been implicated in osteoarthritis and osteoporosis, but disease-modifying evidence remains preliminary and heterogeneous [76,78] (Figure 3). Collectively, these observations emphasize the need for organ- and disease-specific strategies, stratification by baseline copper status, and cautious modulation that avoids iatrogenic deficiency in older adults.

**Figure 4:**
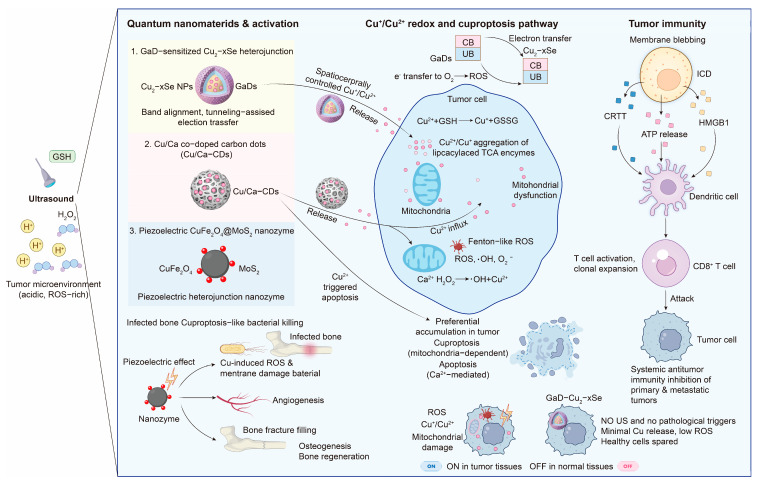
Quantum-enabled nanomaterials for cuproptosis-based cancer intervention and tumor immunity. Quantum nanomaterials enable controlled Cu^+^ and Cu^2+^ redox cycling and ROS generation in the tumor microenvironment to trigger mitochondrial stress and cuproptosis. Tumor cell damage can promote immunogenic signaling and dendritic cell activation, followed by cluster of differentiation 8 (CD8^+^) T cell responses while limiting off-target exposure in normal tissues. Abbreviations: ROS, reactive oxygen species; Cu^+^, cuprous ion; Cu^2+^, cupric ion; CD8^+^, cluster of differentiation 8-positive.

Several precision-delivery concepts are being explored to improve selectivity and reduce systemic exposure, which is appealing for geriatric translation, where tolerability often limits dosing. Nanotechnology-enabled platforms aim to concentrate copper modulation in diseased tissue and enable cue-triggered release in response to local features, such as acidity or oxidative stress [106]. Proposed designs include copper-containing nanoparticles, metal–organic frameworks, and biomimetic carriers engineered for preferential accumulation and controlled release [107,108] (Figure 4). At present, these approaches remain investigational. Their potential clinical value in older adults will be decided by reproducible control over copper speciation and dosing, predictable pharmacokinetics and biodistribution, and convincing long-term safety profiles [109,110,111]. Until these requirements are met, precision delivery should be presented as a promising direction rather than a near-term geriatric solution.

Safety constraints and systemic copper perturbation risks should be integrated into therapeutic discussions of cuproptosis induction. Beyond induction efficacy, copper-manipulating regimens require predefined tolerability endpoints, including longitudinal assessment of body weight and clinical condition, liver and renal function, and hematologic indices, with organ histopathology when feasible [112]. Systemic risk is shaped by both the magnitude and the distribution of copper exposure, and off-target enrichment in circulating or hepatic compartments can narrow the therapeutic window and increase toxicity risk. Accordingly, induction-oriented platforms should prioritize delivery designs that restrict copper stress to diseased tissues and incorporate biomarker-informed monitoring of systemic copper pools with conservative stopping rules to reduce off-target organ injury and iatrogenic copper deficiency [13].

Overall, in older adults, clinical feasibility is primarily determined by tolerability, monitoring capacity, and avoidance of iatrogenic copper deficiency. In the near term, the strongest translational case remains oncology, where copper ionophores and copper depletion strategies can be integrated as sensitizing components within combination regimens, provided that dosing and sequencing are explicit and cumulative toxicity is tracked over time. Outside cancer, evidence remains largely preclinical and heterogeneous, supporting a need for stratification by baseline copper status and inflammatory milieu and for endpoints that prioritize functional preservation in aged tissues [16,95].

## 6. Future Perspectives and Challenges

Despite rapid progress in defining the molecular framework of cuproptosis, several barriers limit translation to age-related diseases. In aging tissues, copper dyshomeostasis, mitochondrial vulnerability, and immune remodeling often co-occur and vary by organ, making it difficult to separate driver effects from secondary amplification. This heterogeneity also means that a single “cuproptosis signature” is unlikely to be universally informative across late-life conditions.

A second barrier is the lack of validated, aging-relevant biomarkers. Much of the current evidence is hypothesis-generating for aging biology because it relies on transcriptional signatures or acute perturbation models, whereas aging reflects slow, cumulative shifts in copper handling and immune competence. Progress will depend on longitudinal, age-stratified studies that integrate systemic copper status, mitochondrial metabolic capacity, and immune phenotypes, with endpoints that link mechanism to clinically meaningful trajectories.

Feasibility in elderly populations remains insufficiently defined because most cuproptosis induction studies are not performed in aged or age-stratified settings, even though older adults typically have a narrower physiological reserve and a higher burden of comorbidity and polypharmacy. Aging-related copper dyshomeostasis is also likely to be heterogeneous across individuals and organs, which argues for biomarker-guided stratification rather than uniform copper modulation. Therefore, future studies should incorporate age-stratified pharmacokinetics and copper biodistribution, together with frailty-relevant tolerability endpoints, such as weight loss and functional decline, and routine monitoring of hepatic and renal reserve and hematologic safety. These data are needed to define geriatric appropriate dosing windows and stopping rules that preserve copper essential functions while minimizing off-target injury.

Therapeutically, a central unresolved issue is directionality. In cancer and other hyperproliferative states, controlled induction of cuproptosis may be advantageous, whereas in degenerative or inflammatory diseases, the priority may be to raise the threshold for copper-induced mitochondrial collapse. The complexity introduced by aging is that often both immune clearance and tissue repair fail. Thus, excessive or misdirected copper metabolism interventions risk promoting tissue loss or chronic inflammation rather than homeostasis. Aging-appropriate therapeutic windows, dosing, and monitoring scenarios will be fundamentally distinct from those developed for younger individuals.

## 7. Conclusions

Cuproptosis links copper dyshomeostasis to mitochondria-centered proteotoxic stress. Aging increases the likelihood that copper stress becomes pathogenic because copper trafficking, redox buffering, mitochondrial quality control, and immune homeostasis progressively drift from their physiological set points.

Across neurodegenerative, cardiovascular, metabolic, musculoskeletal, and hereditary copper-handling disorders, the literature reviewed here supports context-dependent associations between copper imbalance and cuproptosis-related signals. At the same time, a comparative reading of available studies shows an uneven evidence base. Many reports rely on cuproptosis-related gene signatures or correlative copper measurements, whereas direct causal testing with cell-type resolution and aged in vivo validation remains limited in several settings. Closing this gap is essential for deciding when cuproptosis is a driver rather than a bystander and for distinguishing it from overlapping regulated cell death programs.

From a translational perspective, the priority in older adults is to control the negative consequences of cuproptosis while preserving copper’s essential physiological functions. Therapeutic strategies should, therefore, be framed by indication-specific directionality and geriatric safety constraints. In oncology, copper modulation may be used to exploit copper dependency and mitochondrial vulnerability in selected patients, but it requires careful dose titration, monitoring, and attention to comorbidities and polypharmacy. In non-malignant aging diseases, where unintended tissue loss is a major concern, the goal is often to raise the threshold for copper-triggered mitochondrial collapse rather than to force cell death, for example, by restoring copper buffering capacity, limiting expansion of labile copper pools, and improving mitochondrial resilience. Across indications, minimizing iatrogenic copper deficiency and avoiding sustained sterile inflammation should be treated as core endpoints, alongside biomarker development that integrates copper status with mitochondrial and immune readouts to define safe therapeutic windows in elderly populations.

## Figures and Tables

**Table 1:** Core regulators and pathways linked to cuproptosis and copper homeostasis in aging and age-related diseases.

Factor	Regulatory Role	Main Mechanism of Action
Ferredoxin 1 (FDX1)	Positive	Reduces Cu^2+^ to Cu^+^ and promotes protein lipoylation [19]
Lipoic acid synthetase (LIAS)	Positive	Catalyzes protein lipoylation [16]
Lipoyltransferase 1 (LIPT1)	Positive	Transfers lipoic acid to target proteins [12]
Dihydrolipoamide dehydrogenase (DLD)	Positive	Supports TCA cycle and protein lipoylation [11]
Dihydrolipoamide S-acetyltransferase (DLAT)	Positive	Lipoylated target for copper-induced aggregation [10]
Pyruvate dehydrogenase E1 subunit alpha 1 (PDHA1)	Positive	Supports TCA cycle and protein lipoylation [11]
Pyruvate dehydrogenase E1 subunit beta (PDHB)	Positive	Maintains PDH complex activity [10]
Solute carrier family 31 member 1 (SLC31A1; CTR1)	Positive	Imports copper into cells [20]
Cytochrome c oxidase copper chaperone (COX17)	Positive	Delivers copper to mitochondria [14]
Metal regulatory transcription factor 1 (MTF1)	Negative	Enhances metal detoxification response [11]
Glutaminase (GLS)	Negative	Alters glutamine metabolism to reduce cuproptosis sensitivity [11]
Cyclin-dependent kinase inhibitor 2A (CDKN2A)	Negative	Modulates cell cycle and stress response [12]
ATPase copper transporting alpha (ATP7A)	Negative	Mediates copper excretion [17]
ATPase copper transporting beta (ATP7B)	Negative	Mediates copper excretion [17]
COMM domain-containing protein 1 (COMMD1)	Negative	Promotes copper efflux via ATP7A [18]
Glutathione (GSH)	Negative	Chelates copper to reduce toxicity [10]

**Table 2:** Disease contexts linked to copper and cuproptosis-relevant mechanisms: neurological exemplars (AD, PD, ALS, HD, stroke, and Menkes disease) with representative pathways (e.g., Aβ-Cu complexes, α-synuclein aggregation, SOD1-Cu binding, and post-stroke copper accumulation).

Disease	Disease-Specific Mechanisms
Alzheimer’s Disease (AD)	Amyloid-β (Aβ)/Cu complex-induced Aβ deposition [54] Tau hyperphosphorylation/aggregation [55] Cu-activated microglial NF-κB signaling [54]
Parkinson’s Disease (PD)	Cu-induced α-synuclein aggregation and Lewy body formation [55] Reduced nigral copper increases dopaminergic neuron vulnerability [55]
Amyotrophic Lateral Sclerosis (ALS)	Superoxide dismutase 1 (SOD1) mutation impairs Cu binding, causes motor neuron degeneration [13]
Huntington’s Disease (HD)	Cu promotes mutant huntingtin (HTT) aggregation [56]
Stroke Menkes disease	Post-stroke copper accumulation triggers cuproptosis via TCA acylated proteins [52] ATP7A loss-of-function disrupts copper distribution and causes brain copper deficiency [80] Mitochondrial respiratory dysfunction contributes to neurodegeneration [81]

**Table 3:** FDA-approved agents that directly modify copper exposure with indirect implications for cuproptosis.

Primary Mechanism	FDA-Approved Drug/Product	Use Context	One-Line Mechanism	Chemical Structures
Copper replacement	Copper histidinate injection (Zycubo)	Pediatric Menkes disease	Subcutaneous copper replacement to restore bioavailable copper in a genetically copper-deficient state	Copper(II)–histidine complex
Block intestinal copper absorption	Zinc acetate (Galzin)	Wilson’s disease maintenance	Induces intestinal metallothionein to trap dietary copper and reduce systemic copper input [98]	Zinc acetate
Intravenous copper supplementation	Cupric chloride injection	Parenteral nutrition (PN/TPN) copper supplementation	Provides intravenous copper to prevent or correct copper deficiency during PN [99,100]	Cupric chloride (CuCl_2_)
Copper-containing parenteral trace-element products	Tralement	PN for adults and pediatric patients ≥ 10 kg	Fixed-dose multi-trace formulation supplying copper and other trace elements for PN requirements [100]	Multi-ingredient product
Copper-containing parenteral trace-element products	Multrys	PN for neonates and pediatric patients < 10 kg	Fixed-dose multi-trace formulation with lower copper content tailored to small pediatric PN [100]	Multi-ingredient product

## Data Availability

No new data were created or analyzed in this study. Data sharing is not applicable to this article.

## Abbreviations

AD	Alzheimer’s disease
ALS	amyotrophic lateral sclerosis
CcO	cytochrome c oxidase
CTR1	copper transporter 1
DAMPs	damage-associated molecular patterns
Fe-S	iron–sulfur
GPX4	glutathione peroxidase 4
GSH	glutathione
HD	Huntington’s disease
IL-6	interleukin-6
MAPK	mitogen-activated protein kinase
mTOR	mechanistic target of rapamycin
NF-κB	nuclear factor kappa B
NLRP3	NLR family pyrin domain containing 3
Nrf2	nuclear factor erythroid 2-related factor 2
OXPHOS	oxidative phosphorylation
PD	Parkinson’s disease
PD-1	programmed cell death protein 1
PD-L1	programmed death-ligand 1
RCD	regulated cell death
ROS	reactive oxygen species
SASP	senescence-associated secretory phenotype
TCA	tricarboxylic acid
TCR	T cell receptor
TLR4	Toll-like receptor 4
TNF-α	tumor necrosis factor-α
TTM	tetrathiomolybdate
